# Preoperative smoking and robot-assisted radical cystectomy outcomes & complications in multicenter KORARC database

**DOI:** 10.1038/s41598-024-61005-6

**Published:** 2024-05-08

**Authors:** Joongwon Choi, Jooyoung Lee, Yu Been Hwang, Byong Chang Jeong, Sangchul Lee, Ja Hyeon Ku, Jong Kil Nam, Wansuk Kim, Ji Youl Lee, Sung Hoo Hong, Koon Ho Rha, Woong Kyu Han, Won Sik Ham, Sung Gu Kang, Seok Ho Kang, Jong Jin Oh, Young Goo Lee, Tae Gyun Kwon, Tae-Hwan Kim, Seung Hyun Jeon, Sang Hyub Lee, Sung Yul Park, Young Eun Yoon, Yong Seong Lee

**Affiliations:** 1 Department of Urology, Chung-Ang University Gwangmyeong Hospital, Chung-Ang University College of Medicine, Gwangmyeong, Korea; 2 Department of Applied Statistics, Chung-Ang University, Seoul, Korea; 3 Department of Urology, Samsung Medical Center, Sungkyunkwan University School of Medicine, Seoul, Korea; 4 Department of Urology, Seoul National University Bundang Hospital, Seongnam, Korea; 5 Department of Urology, Seoul National University College of Medicine, Seoul, Korea; 6 Department of Urology, Pusan National University Yangsan Hospital, Yangsan, Korea; 7 Department of Urology, Ewha Womans University Mokdong Hospital, Seoul, Korea; 8 Department of Urology, Seoul St. Mary’s Hospital, The Catholic University of Korea College of Medicine, Seoul, Korea; 9 Department of Urology, Severance Hospital, Yonsei University College of Medicine, Seoul, Korea; 10 Department of Urology, Korea University College of Medicine, Seoul, Korea; 11 Department of Urology, Hallym University School of Medicine, Seoul, Korea; 12 Department of Urology, Kyungpook National University School of Medicine, Daegu, Korea; 13 Department of Urology, KyungHee University College of Medicine, Seoul, Korea; 14 Department of Urology, Hanyang University College of Medicine, Seoul, Korea

**Keywords:** Bladder cancer, Smoking, Cystectomy, Survival analysis, Robot-assisted surgery, Urological cancer, Bladder cancer, Bladder

## Abstract

To investigate the influence of preoperative smoking history on the survival outcomes and complications in a cohort from a large multicenter database. Many patients who undergo radical cystectomy (RC) have a history of smoking; however, the direct association between preoperative smoking history and survival outcomes and complications in patients with muscle-invasive bladder cancer (MIBC) who undergo robot-assisted radical cystectomy (RARC) remains unexplored. We conducted a retrospective analysis using data from 749 patients in the Korean Robot-Assisted Radical Cystectomy Study Group (KORARC) database, with an average follow-up duration of 30.8 months. The cohort was divided into two groups: smokers (n = 351) and non-smokers (n = 398). Propensity score matching was employed to address differences in sample size and baseline demographics between the two groups (n = 274, each). Comparative analyses included assessments of oncological outcomes and complications. After matching, smoking did not significantly affect the overall complication rate (*p* = 0.121). Preoperative smoking did not significantly increase the occurrence of complications based on complication type (p = 0.322), nor did it increase the readmission rate (p = 0.076). There were no perioperative death in either group. Furthermore, preoperative smoking history showed no significant impact on overall survival (OS) [hazard ratio (HR) = 0.87, interquartile range (IQR): 0.54–1.42; *p* = 0.589] and recurrence-free survival (RFS) (HR = 1.12, IQR: 0.83–1.53; *p* = 0.458) following RARC for MIBC. The extent of preoperative smoking (≤ 10, 10–30, and ≥ 30 pack-years) had no significant influence on OS and RFS in any of the categories (all *p* > 0.05). Preoperative smoking history did not significantly affect OS, RFS, or complications in patients with MIBC undergoing RARC.

## Introduction

Bladder cancer poses a significant public health challenge, contributing significantly to global mortality and morbidity. Bladder cancer is the 10th most prevalent cancer globally, with 573,278 new cases and over 200,000 deaths in 2020^1^.

The primary cause of bladder cancer incidence is smoking, with a substantial proportion of patients who undergo radical cystectomy (RC) being current or former smokers^2^. The complex composition of tobacco smoke, comprising over 60 carcinogens linked to at least 18 types of cancer, makes smoking the second leading risk factor for mortality^3^. Cigarette smoking has been established as the most significant risk factor, increasing the risk of bladder cancer by up to fivefold^4^.

Preoperative smoking history correlates with a high tumor stage and grade in patients with newly diagnosed urothelial carcinoma of the bladder, affecting both smoking status and quantity^3^. This influence extends to non-muscle invasive bladder cancer (NMIBC), affecting disease recurrence and progression^5,6^.

Although the correlation between smoking and bladder cancer incidence is well-established, its impact on cancer recurrence following RC in patients with muscle-invasive bladder cancer (MIBC) has been highlighted^3^. RC, a complex surgical procedure with a complication rate of 30–64%^7^, presents post-RC patients who smoke with an increased risk of perioperative complications and the need for re-operative surgery^8,9^. However, the direct association between preoperative smoking history and survival outcomes and complications in patients with MIBC undergoing robot-assisted radical cystectomy (RARC) remains insufficiently explored.

The Korean Robot-Assisted Radical Cystectomy Study Group (KORARC) database, a multicenter repository formed by several institutions, has been instrumental in the development of various bladder cancer-related research outcomes^10–13^. The database comprehensively documents detailed preoperative smoking history, including pack-years.

In this study, we hypothesized that preoperative smoking may impact survival and contribute to wound or infection complications in RARC patients. Consequently, we aimed to investigate the influence of preoperative smoking history on survival outcomes and complications in a cohort derived from a large multicenter database.

## Patients and methods

### Data acquisition & analysis

A retrospective analysis was conducted using data from 749 patients enrolled in the Korean Robot-Assisted Radical Cystectomy Study Group (KORARC) database. RARC was performed at 11 tertiary referral centers (21 surgeons) in the Republic of Korea between April 2007 and November 2019. The primary data source was the Korean Society of Endourology and Robotics. The cohort with an average follow-up of 30.8 months, was divided into two groups: 351 smokers and 398 non-smokers. Smokers included both current smokers (those who were actively smoking up to the time of RARC) and ex-smokers with a preoperative smoking history, while non-smokers were defined as individuals who have never smoked.

Propensity score matching was used to address significant differences in sample size and baseline demographics between the two groups. Comparative analyses included demographics, preoperative treatment, intraoperative outcomes, oncological outcomes, and complications (n = 274, each). The study was conducted in accordance with the Declaration of Helsinki and was approved by the Institutional Review Board of Korea University Anam Hospital (approval number: 2019AN0102). Written informed consent was obtained from all participants.

### Statistical analysis

Demographic and clinical characteristics were presented as median and interquartile ranges (IQRs) for continuous data and as numbers and percentages for categorical data. Each patient in the preoperative smoking group was matched with a propensity score, using the nearest-neighbor method, to one individual in the preoperative non-smoking group. A logistic regression model was used for the propensity score model, including age, sex, height, weight, body mass index (BMI), American Society of Anesthesiologists (ASA) score, history of preoperative abdominal surgery, history of complications (cardiac, pulmonary, and renal), history of medical conditions (hypertension, diabetes mellitus, hyperlipidemia, and benign prostate hyperplasia), and family history of bladder cancer. Standard mean differences (SMDs) were used to assess the covariate balance between the preoperative smoking and non-smoking groups following propensity score matching. The distribution of confounders was balanced between the two groups when the SMDs were < 10%. Crude incidence rates per 1000 person-years were calculated for overall survival (OS) and recurrence-free survival (RFS). Kaplan–Meier estimates were used for OS and RFS, stratified by preoperative smoking status.

Using propensity score-matched data, hazard ratios (HRs) and 95% confidence intervals (CIs) for each outcome were estimated using Cox proportional hazard regression models, and robust standard errors were used to determine the correlation between matching pairs. Moreover, logistic regression and multinomial logistic regression models were used to estimate the odds ratios (ORs) and 95% CIs for the incidence, types, and dates of complications using the matched data.

Subgroup analyses were performed according to age (< 65 and ≥ 65 years). We assessed the effect of pack-years of smoking as continuous and categorical variables (≤ 10, 10–30, and ≥ 30 pack-years of smoking) and on OS and RFS in preoperative smokers using Cox regression models after adjusting for the year of operation, age, sex, height, weight, BMI, ASA, history of preoperative abdominal surgery, history of complications (cardiac, pulmonary, renal), history of medical conditions (hypertension, diabetes mellitus, hyperlipidemia, and benign prostate hyperplasia), and family history of bladder cancer.

We also accessed that achieving pentafecta, composed of negative soft tissue surgical margin, removal of ≥ 16 lymph nodes, no major complications (Clavien–Dindo grade 3–5) within 90 days, no clinical recurrence within the first 12 months, and no ureteroenteric stricture, from previous studies^10^, impacts patients' OS and PFS. We investigated whether there was a difference in pentafecta achievement between the smoker and non-smoker groups.

Statistical significance was set at a *p* > 0.05 with two-sided tests. All statistical analyses were performed using R version 4.3.1 (The R Foundation for Statistical Computing, Vienna, Austria).

## Results

The baseline characteristics based on smoking history before and after propensity score matching are presented in Table 1. Before matching, statistically significant differences were found in age, sex, BMI, history of preoperative abdominal surgery, history of benign prostate hyperplasia and diversion type. Before propensity matching, a higher proportion of neobladder was observed in the smoker group (55.3% vs 48.7%, p = 0.029), which may be attributed to the likelihood of a younger patients being included in the smoker group (65 vs 67, p = 0.011). However, after matching, these variables were successfully corrected.

**Table 1:** Baseline characteristics by smoking history before and after propensity score matching.

	Before propensity matching			After matching		
	Smoker (n = 351)	Non-smoker (n = 398)	P value	Smoker (n = 274)	Non-smoker (n = 274)	P value
Age, median (IQR)	65 (57—72)	67 (59—74)	0.011	65 (57—72)	65 (58—73)	0.347
Sex, n (%)			< 0.001			1
Women	3 (0.9)	109 (27.4)		3 (1.1)	3 (1.1)	
Men	348 (99.1)	289 (72.6)		271 (98.9)	271 (98.9)	
Height, median (IQR)	167.7 (163—172)	164 (158—170)	< 0.001	167.6 (163—172)	167.3 (163–171)	0.427
Weight, median (IQR)	67.6 (61.2—74.0)	65.0 (56.9—72.3)	< 0.001	67.0 (60.5—73.5)	67.5 (60.0–75.0)	0.877
BMI, median (IQR)	24.2 (22.4—26.0)	24.05 (22.0—26.4)	0.807	24 (22.3–25.9)	24.05 (22.1—26.4)	0.544
ASA, n (%)	2 (1—2)	2 (1—2)	0.415	2 (1—2)	2 (1—2)	0.772
Cardiac	33 (9.4)	44 (11.1)	0.533	28 (10.2)	27 (9.9)	1
Pulmonary	27 (7.7)	24 (6.0)	0.450	18 (6.6)	16 (5.8)	0.859
Renal	55 (15.7)	66 (16.6)	0.811	39 (14.2)	44 (16.1)	0.634
HTN	153 (43.6)	171 (43.0)	0.922	115 (42.0)	118 (43.1)	0.863
DM	83 (23.6)	92 (23.1)	0.932	58 (21.2)	65 (23.7)	0.539
Hyperlipidemia	25 (7.1)	32 (8.0)	0.738	19 (6.9)	19 (6.9)	1
BPH	49 (14.0)	27 (6.8)	0.002	39 (14.2)	27 (9.9)	0.149
Abdominal surgery history, n (%)	71 (20.2)	109 (27.4)	0.028	60 (21.9)	57 (20.8)	0.835
Abdominal radiation history, n (%)	13 (3.7)	11 (2.8)	0.602	11 (4.0)	8 (2.9)	0.641
Diversion type, n (%)			0.029			0.108
Neobladder	194 (55.3)	194 (48.7)		147 (53.6)	144 (52.6)	
Conduit	128 (36.5)	148 (37.2)		104 (38.0)	92 (33.6)	
Etc	29 (8.3)	56 (14.1)		23 (8.4)	38 (13.9)	

*BMI* body mass index, *ASA* American Society of Anesthesiologists score, *HTN* hypertension, *DM* diabetes mellitus, *BPH* benign prostate hyperplasia.

Postoperative complications were thoroughly assessed to determine whether preoperative smoking history influenced complication rates (Table 2). Preoperative smoking did not significantly increase the occurrence of complications based on complication type (*p* = 0.322), nor did it increase the readmission rate (p = 0.076). Additionally, upon examining the Clavien-Dindo grade, smoking did not increase severe complications of grade 3 or higher (49 vs 59, *p* = 0.173). There were no perioperative death in either group.

**Table 2:** Type and Clavien-Dindo grade of complications after matching.

	Smoker (n = 274)	Non-smoker (n = 274)	P value
Complication type, n (%)			0.322
Gastrointestinal	37 (25.2)	54 (32.7)	
Hematological	5 (3.4)	3 (1.8)	
Genitourinary	33 (22.4)	37 (22.4)	
Cardiovascular	2 (1.4)	2 (1.2)	
Pulmonary	1 (0.7)	5 (3.0)	
Infection	36 (24.5)	36 (21.8)	
Wound	13 (8.8)	5 (3.0)	
Neurological	2 (1.4)	3 (1.8)	
Vascular	5 (3.4)	3 (1.8)	
Dermatological	0 (0.0)	0 (0.0)	
Etc	13 (8.8)	17 (10.3)	
Readmission rate, n (%)	89 (32.5)	110 (40.1)	0.076
Clavien-Dindo grade, n (%)			0.173
No complication	127 (46.4)	110 (40.1)	
1	17 (6.2)	11 (4.0)	
2	81 (29.6)	94 (34.3)	
3A	21 (7.7)	15 (5.5)	
3B	26 (9.5)	38 (13.9)	
4A	2 (0.7)	5 (1.8)	
4B	0 (0.0)	1 (0.4)	
5 (death)	0 (0.0)	0 (0.0)	

In logistic and multinomial logistic regression analysis, we presented whether preoperative smoking increases the risk of complications, particularly infection and wound complications in Table 3. Smoking did not significantly affect overall complication rates (*p* = 0.121). When categorized by type, a preoperative smoking history did not influence infection, wound complications, or other complications (*p* > 0.05).

**Table 3:** Logistic and multinomial logistic regression analyses for the effect of preoperative smoking on complications, the types of complications, and the occurrence of complications by dates.

Outcomes	OR	95% CI	P value
Complications	0.77	0.54–1.07	0.121
Complication types			
Infection and wound	1.03	0.63–1.67	0.919
Others	0.68	0.47–0.98	0.039
Complication dates			
Within 30 days	0.67	0.45–1.00	0.053
Within 30–90 days	0.88	0.58–1.34	0.555
After 90 days	0.86	0.05–13.92	0.916

Furthermore, an investigation was conducted to ascertain whether postoperative complications were influenced by smoking status and age (Supplementary Table 3). Comparing patients who were < 65 years with those who were ≥ 65 years, we found that, excluding infection and wound complications, the rate of other complications within 30 days was lower in the < 65 years group (OR = 0.49, 0.28–0.85; *p* = 0.012). Regardless of age, a preoperative smoking history did not have a significant impact on complications within 30 days, 30–90 days, or beyond 90 days (*p* > 0.05).

We also exploring the impact of the extent of preoperative smoking (≤ 10, 10–30, ≥ 30 pack-years) on OS and RFS revealed that smoking had no significant influence on any of the categories (Table 4, *p* > 0.05). Whether there is a difference in pentafecta achievement between the two groups is described in Table 5. There was no statistically significant difference in pentafecta achievement based on preoperative smoking status and the five-component of pentafecta (p = 0.122).

**Table 4:** Hazard ratios from Cox models for the effects of pack-years of smoking on overall survival and recurrence-free survival.

	HR (95% CI)	P value
Overall survival		
≤ 10 pack-years of smoking	1 (reference)	
10–30 pack-years of smoking	0.93 (0.12, 6.95)	0.945
≥ 30 pack-years of smoking	0.57 (0.1, 3.39)	0.535
Recurrence-free survival		
≤ 10 pack-years of smoking	1 (reference)	
10–30 pack-years of smoking	1.61 (0.43, 5.99)	0.474
≥ 30 pack-years of smoking	2.23 (0.66, 7.54)	0.198
Overall survival	0.97 (0.94, 1.01)	0.108
Recurrence-free survival	1.01 (0.99, 1.03)	0.199

Adjusted for OP year, age, sex, height, weight, BMI, ASA, PRE abdominal surgery history, complication (cardiac, pulmonary, renal, HTN, DM, Hyperlipidemia, BPH), family history.

**Table 5:** Pentafecta achievement after matching.

	Smoker (n = 274)	Non-smoker (n = 274)	P value
Pentafecta achievement (%)	99 (36.1)	81 (29.6)	0.122
Negative soft tissue surgical margin	261 (95.3)	265 (96.7)	0.514
≥ 16 lymph nodes removed	154 (56.2)	142 (51.8)	0.346
No major complications (Clavien–Dindo grade 3–5) within 90 days	225 (82.1)	215 (78.5)	0.334
No clinical recurrence within the first 12 months	221 (80.7)	230 (83.9)	0.371
No ureteroenteric stricture	252 (92.0)	246 (89.8)	0.458

**Figure 1:**
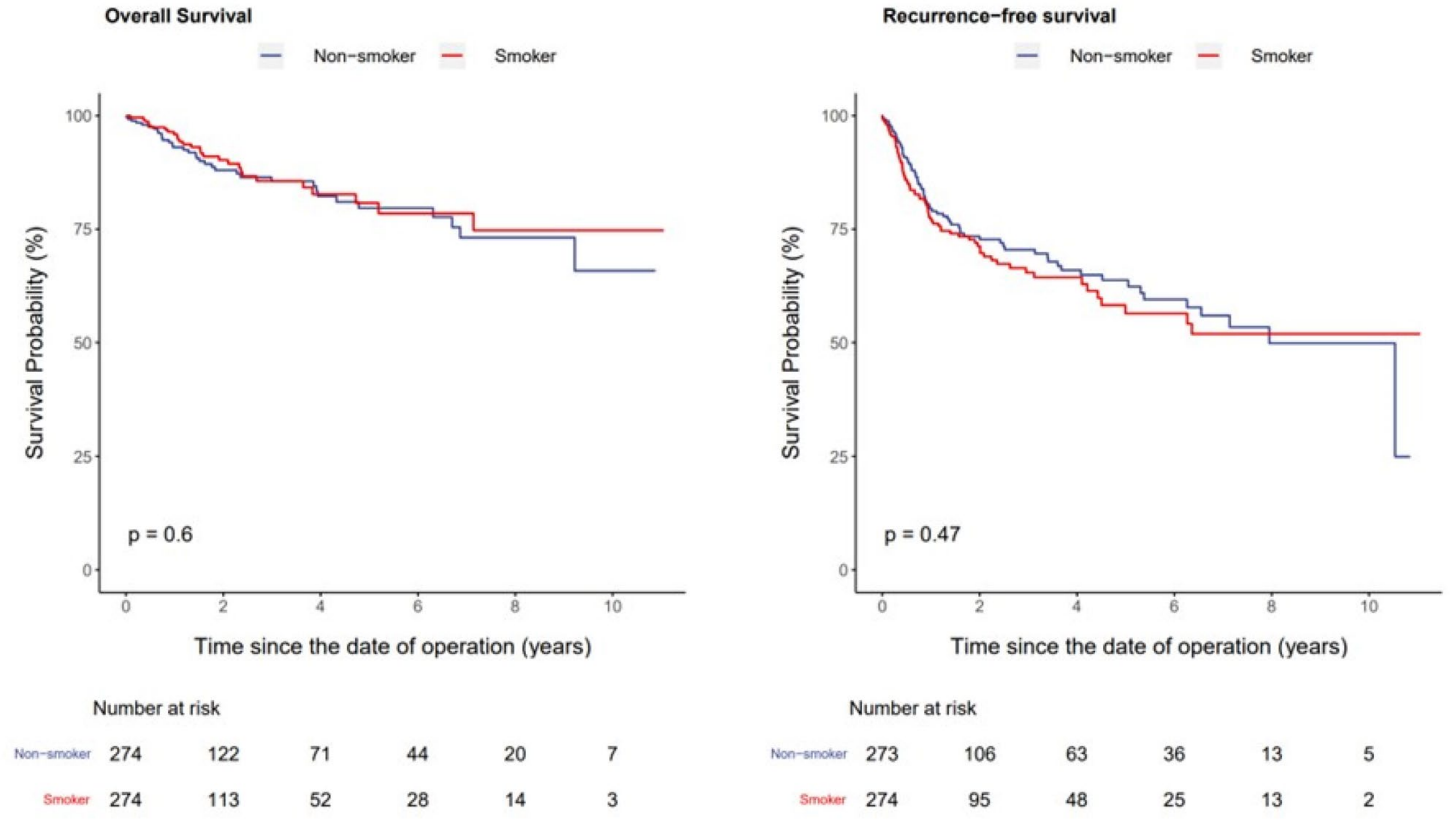
Kaplan–Meier curves. Kaplan–Meier curves for overall and recurrence-free survival according to smoking status.

The incidence rates per 1000 person-years and HRs from the Cox models for each outcome comparing the smoker and non-smoker groups are shown in Supplementary Table 1. After matching, preoperative smoking history did not significantly impact OS (HR = 0.87, IQR: 0.54–1.42; *p* = 0.589) and RFS (HR = 1.12, IQR: 0.83–1.53; *p* = 0.458) after RARC for MIBC. A detailed Kaplan–Meier curve is shown in Fig. 1.

To investigate whether age influences survival due to preoperative smoking history, a survival comparison was conducted by categorizing patients into groups based on age, namely < 65 years and ≥ 65 years (Supplementary Table 2). Regardless of age group, preoperative smoking history did not affect OS or RFS (all *p* > 0.05).

## Discussion

In our study, preoperative smoking history before RARC did not affect patient survival or postoperative complications. This contradicts previous findings that smoking influences RC outcomes. However, further research is warranted to interpret these results due to the large-volume multicenter database and propensity score matching employed in our investigation.

We conducted various subgroup analyses, comparing survival, smoking frequency, age, and early and late postoperative complications. However, no significant differences were observed between the two groups. Given the higher likelihood of postoperative complications in patients who undergo radical cystectomy than in those who undergo other surgeries, multidisciplinary discussions are essential, and postoperative care must be comprehensive to effectively manage risk factors such as smoking status^1,14,15^.

Approximately half of bladder cancer cases are attributed to tobacco smoking^16^. For patients undergoing RARC, physicians should strongly recommend smoking cessation. While unable to reflect postoperative smoking status, our study affirms that preoperative smoking history does not affect OS and RFS in patients with RARC. These results suggest that smoking cessation after surgery may potentially have a more significant impact on survival.

RARC is associated with reduced incisions and lower blood loss compared with open RC, possibly resulting in fewer postoperative complications. Additionally, the feasibility of RARC has been reported in T4 cases and is not limited to T3 or below^17^. Therefore, preoperative factors, such as smoking, may be less likely to have an impact on RARC different from that on open RC. Previous studies have reported an increased Clavien III-V complication rate in current smokers following RC (13.1% vs. 7.4%)^9^. Our study, focusing on patients undergoing RARC, specifically investigated the influence of preoperative smoking history and found distinctions. Furthermore, the lower incidence of complications seen in our study may be attributable to age, as the average age in our study was 65 years, whereas it was 69 years in the previous study. Particularly, smoking status has been reported to influence complications in older patients aged ≥ 70 years^18^. However, in our study, focusing on RARC patients ≥ 65 years, no statistically significant differences were found.

In the STOP-OP randomized controlled trial, a preoperative 6-week intensive smoking and/or alcohol cessation intervention was conducted to assess its impact on postoperative complications following RC. However, the intervention did not influence postoperative complications following radical cystectomy^7^. A previous meta-analysis suggested that smoking status is associated with an increased risk of major postoperative complications, infections, and mortality after RC; however, most of the data were from patients who underwent open RC^19^. A previous study reported a controversial relationship between smoking and RC, where current smoking status had a selection effect, and no adverse bladder cancer-related outcomes were present among current smokers after RC^20^.

Pelvic radiotherapy can also have a significant impact on post-radical cystectomy complications. In our study, although there was no difference in the rate of radiation therapy between the two groups (4.0% vs 2.9%, *p* = 0.641), a study involving 682 patients investigating the influence of radiation therapy reported that 75.1% of patients with pelvic radiotherapy experienced complications after radical cystectomy. Furthermore, urinary tract infection (19%) was reported to be the most common complication, emphasizing the need for careful post-operative management in such patients^21^.

Venous thromboembolism (VTE) is another rare but significant complication associated with radical cystectomy that can increase perioperative mortality. Upon examining our data, we found three cases of VTE events, with two cases classified as Clavien grade 2 and one case as grade 3. In a large-scale study on VTE following radical cystectomy, a VTE incidence rate of 4% was reported, with 24% resulting in fatalities, and the median time to VTE occurrence was reported to be 11 days post-surgery^22^.

Our study had several limitations. First, the study was retrospective in nature. Smoking status was only assessed preoperatively; information regarding postoperative smoking cessation was not investigated. Therefore, the results of this study should be interpreted from the perspective of preoperative smoking status. Second, the database lacked details regarding he time at which ex-smokers ceased smoking; it only provided the total quantity smoked. Consequently, differentiating between individuals who had quit smoking 10 years prior and those who had quit a month ago was not feasible. Third, due to the limited number of current smokers in this study, we were unable to analyze current smokers and ex-smokers separately. Fourth, we investigated only the presence of medical diseases as categorical data and did not collect data on laboratory parameters such as renal and hepatic function. Concurrent comorbidities associated with this may also potentially influence complication rates.

Nonetheless, the significance of these data lies in assessing the impact of preoperative smoking history within a more uniform patient population, focusing solely on the outcomes of patients undergoing RARC.

## Conclusion

In conclusion, the preoperative smoking history of patients with MIBC did not exhibit any significant impacts on OS, RFS, or complications in patients who underwent RARC.

### Supplementary Information

Supplementary Tables.

## Data Availability

The datasets generated and/or analyzed in the current study are available from the corresponding author upon reasonable request.

## References

[1] Tfaily MA, Tamim H, El Hajj A, Mukherji D (2022). Muscle invasive bladder cancer and radical cystectomy: A risk predictive model. Ecancermedicalscience..

[2] Beech BB, Doudt AD, Sjoberg DD (2023). Association of smoking history on health-related quality of life in patients undergoing radical cystecomy. Urol. Oncol..

[3] Rink M, Zabor EC, Furberg H (2013). Impact of smoking and smoking cessation on outcomes in bladder cancer patients treated with radical cystectomy. Eur. Urol..

[4] Freedman ND, Silverman DT, Hollenbeck AR, Schatzkin A, Abnet CC (2011). Association between smoking and risk of bladder cancer among men and women. JAMA.

[5] Chen CH, Shun CT, Huang KH (2007). Stopping smoking might reduce tumour recurrence in nonmuscle-invasive bladder cancer. BJU Int..

[6] Ogihara K, Kikuchi E, Yuge K (2016). Refraining from smoking for 15 years or more reduced the risk of tumor recurrence in non-muscle invasive bladder cancer patients. Ann. Surg. Oncol..

[7] Lauridsen SV, Thomsen T, Jensen JB (2022). Effect of a smoking and alcohol cessation intervention initiated shortly before radical cystectomy—The STOP-OP Study: A randomised clinical trial. Eur. Urol. Focus.

[8] Reese SW, Ji E, Paciotti M (2020). Risk factors and reasons for reoperation after radical cystectomy. Urol. Oncol..

[9] Sathianathen NJ, Weight CJ, Jarosek SL, Konety BR (2018). Increased surgical complications in smokers undergoing radical cystectomy. Bladder Cancer..

[10] Oh JJ, Lee S, Ku JH (2021). Oncological outcome according to attainment of pentafecta after robot-assisted radical cystectomy in patients with bladder cancer included in the multicentre KORARC database. BJU Int..

[11] Shim JS, Noh TI, Ku JH (2021). Effect of intraoperative fluid volume on postoperative ileus after robot-assisted radical cystectomy. Sci. Rep..

[12] Jin HJ, Shim JS, Kwon TG (2022). Gender-related outcomes in robot-assisted radical cystectomy: A multi-institutional study. Investig. Clin. Urol..

[13] Kim H, Jeong BC, Lee S (2022). Predicting factor analysis of postoperative complications after robot-assisted radical cystectomy: Multicenter KORARC database study. Int. J. Urol..

[14] Knorr JM, Ericson KJ, Zhang JH (2021). Comparison of major complications at 30 and 90 days following radical cystectomy. Urology..

[15] Boeri L, Soligo M, Frank I (2019). Cigarette smoking is associated with adverse pathological response and increased disease recurrence amongst patients with muscle-invasive bladder cancer treated with cisplatin-based neoadjuvant chemotherapy and radical cystectomy: A single-centre experience. BJU Int..

[16] Mori K, Mostafaei H, Abufaraj M, Yang L, Egawa S, Shariat SF (2020). Smoking and bladder cancer: Review of the recent literature. Curr. Opin. Urol..

[17] Al-Daghmin A, Kauffman EC, Shi Y (2014). Efficacy of robot-assisted radical cystectomy (RARC) in advanced bladder cancer: Results from the International Radical Cystectomy Consortium (IRCC). BJU Int..

[18] Haeuser L, Marchese M, Schrag D (2021). The impact of smoking on radical cystectomy complications increases in elderly patients. Cancer..

[19] Tellini R, Mari A, Muto G (2021). Impact of smoking habit on perioperative morbidity in patients treated with radical cystectomy for urothelial bladder cancer: A systematic review and meta-analysis. Eur. Urol. Oncol..

[20] Froehner M, Koch R, Hubler M (2018). Selection effects may explain smoking-related outcome differences after radical cystectomy. Eur. Urol. Focus.

[21] Gontero P, Pisano F, Palou J (2020). Complication rate after cystectomy following pelvic radiotherapy: An international, multicenter, retrospective series of 682 cases. World J. Urol..

[22] Laymon M, Harraz A, Elshal A (2019). Venous thromboembolism after radical cystectomy and urinary diversion: A single-center experience with 1737 consecutive patients. Scand. J. Urol..

