# Supplementary material for: Preoperative smoking and robot-assisted radical cystectomy outcomes & complications in multicenter KORARC database
Source: Sci Rep. 2024 May 8;14:10550. doi: 10.1038/s41598-024-61005-6 (PMC11078966; doi:10.1038/s41598-024-61005-6)
Supplement: Supplementary file 1 — Supplementary Tables. [file 41598_2024_61005_MOESM1_ESM.docx]

Supplementary Table 1. Incidence rate per 1000 person years and hazard ratios from Cox models for each outcome comparing smoker and non-smoker groups

| Outcomes | Smoker | | Non-smoker | | Hazard Ratio (95% CI) | P value |
| --- | --- | --- | --- | --- | --- | --- |
|  | Event, No. (%) | Incidence rate  (per 1000 person-years) | Event, No. (%) | Incidence rate  (per 1000 person-years) |  |  |
| Overall survival | 28 | 42.9 (29.6, 62.1) | 35 | 46.3 (33.3, 64.5) | 0.87 (0.54, 1.42) | 0.589 |
| Recurrence-free survival | 78 | 135.1 (108.2, 168.7) | 73 | 111 (88.3, 139.6) | 1.12 (0.83, 1.53) | 0.458 |

Supplementary Table 2. Incidence rate per 1000 person years and hazard ratios from Cox models for each outcome comparing smoker and non-smoker groups according to age

| Outcomes | Smoker | | Non-smoker | | Hazard Ratio (95% CI) | P value |
| --- | --- | --- | --- | --- | --- | --- |
|  | Event, No. (%) | Incidence rate  (per 1000 person-years) | Event, No. (%) | Incidence rate  (per 1000 person-years) |  |  |
| < 65 |  |  |  |  |  |  |
| Overall survival | 12 | 36.2 (20.6, 63.8) | 14 | 33.5 (19.8, 56.5) | 1.04 (0.49, 2.22) | 0.915 |
| Recurrence-free survival | 36 | 122.4 (88.3, 169.7) | 33 | 92.3 (65.6, 129.7) | 1.21 (0.75, 1.95) | 0.442 |
| ≥ 65 |  |  |  |  |  |  |
| Overall survival | 16 | 49.7 (30.5, 81.2) | 21 | 63.4 (40.7, 95.6) | 0.79 (0.41, 1.52) | 0.480 |
| Recurrence-free survival | 42 | 148.3 (109.6, 200.7) | 40 | 133.4 (97.8, 181.8) | 1.09 (0.73, 1.63) | 0.672 |

Supplementary Table 3. Logistic and multinomial logistic regression analyses for the effect of preoperative smoking on complications, the types of complications, and the occurrence of complications by dates according to age

| Outcomes | OR | 95% CI | P value |
| --- | --- | --- | --- |
| Complications |  |  |  |
| Age <65 | 0.64 | 0.39 - 1.06 | 0.086 |
| Age ≥65 | 0.89 | 0.56 – 1.40 | 0.603 |
| Complication types |  |  |  |
| Age <65 |  |  |  |
| Infection + Wound | 1.11 | 0.56 – 2.23 | 0.759 |
| Others | 0.49 | 0.28 -0.85 | 0.012 |
| Age ≥65 |  |  |  |
| Infection + Wound | 0.89 | 0.45 – 1.8 | 0.755 |
| Others | 0.88 | 0.54 – 1.44 | 0.616 |
| Complication date |  |  |  |
| Age <65 |  |  |  |
| Within 30 Days | 0.58 | 0.32 – 1.04 | 0.068 |
| Within 30-90 days | 0.73 | 0.39 – 1.37 | 0.325 |
| After 90 days | 0.73 | 0.04 – 11.98 | 0.826 |
| Age ≥65 |  |  |  |
| Within 30 Days | 0.76 | 0.44 – 1.32 | 0.334 |
| Within 30-90 days | 1.03 | 0.59 – 1.80 | 0.907 |
| After 90 days | - | - | - |
